# Interpersonal gait synchronization reduces gait asymmetry and induces a carry-over effect during overground side-by-side walking

**DOI:** 10.3389/fpsyg.2026.1892679

**Published:** 2026-09-09

**Authors:** Yuntong Lu, Hirotaka Uchitomi, Yoshihiro Miyake

**Affiliations:** Department of Computer Science, School of Computing, Institute of Science Tokyo, Yokohama, Japan

**Keywords:** carry-over effect, gait asymmetry, gait rehabilitation, inertial measurement unit, interpersonal synchronization, side-by-side walking

## Abstract

**Introduction:**

Interpersonal gait synchronization is widely observed during side-by-side walking and has been hypothesized to contribute to gait rehabilitation. Nonetheless, the specific effects of gait synchronization on gait asymmetry, as distinguished from mere side-by-side walking, have not been experimentally investigated under overground walking conditions. This study aimed to examine the effects of interpersonal gait synchronization on gait asymmetry during overground side-by-side walking.

**Methods:**

Ten pairs of participants performed overground side-by-side walking tasks under synchronization and asynchronization (control) conditions. Gait asymmetry was simulated in one member of each pair by applying unilateral ankle loading and was quantified across five spatiotemporal gait parameters using inertial measurement unit sensors. The degree of interpersonal synchronization was verified using the Gait Synchronization Index (GSI). Statistical analyses were conducted using Friedman’s test, followed by Wilcoxon signed-rank test with Bonferroni correction.

**Results:**

All participants achieved synchronized walking under the synchronization condition and asynchronized walking under the asynchronization condition, as verified by the GSI values. Compared with baseline, stride height asymmetry was significantly reduced during synchronized walking (*W* = 0, *p* = 0.002, *r* = 0.979), and this reduction was maintained immediately after synchronization ceased (*p* = 0.002), indicating a short-term carry-over effect. No significant changes in gait asymmetry or other gait parameters were observed under the asynchronization condition.

**Conclusion:**

Interpersonal gait synchronization specifically reduces stride height asymmetry and produces a short-term carry-over effect in overground walking, suggesting its potential as a basis for gait rehabilitation interventions.

## 1 Introduction

Gait asymmetry, defined as a bilateral difference in spatiotemporal gait parameters between the left and right limbs, is a prominent feature of gait dysfunction associated with neurological conditions such as stroke and Parkinson’s disease (PD) (Patterson et al., 2008; Plotnik et al., 2007). Because gait asymmetry is associated with increased fall risk, higher metabolic cost, and reduced walking velocity, reducing gait asymmetry is an important target of gait rehabilitation (Patterson et al., 2008). During gait rehabilitation, therapists generally walk side by side with patients to provide rhythmic guidance, which may lead to interpersonal gait synchronization. Supervised walking with a physiotherapist is reportedly more effective in addressing gait disorders than unsupervised practice (Coulter et al., 2013). Additionally, collaborative walking practice is considered particularly suitable for patients with post-stroke movement disorders (Kaur et al., 2012). These observations suggest that interpersonal gait synchronization may play an active role in gait rehabilitation; nonetheless, the specific effects of gait synchronization on individual gait performance have not yet been fully established.

Interpersonal gait synchronization during side-by-side walking has been widely documented (Zivotofsky and Hausdorff, 2007; Felsberg and Rhea, 2021; Hu et al., 2022). Importantly, synchronization is not an automatic consequence of walking in proximity: even during side-by-side walking, spontaneous synchronization emerges in only about half of trials and depends on the sensory communication available between partners (Zivotofsky and Hausdorff, 2007). Proximity and synchronization are therefore dissociable. Isolating the contribution of synchronization requires manipulating synchronization while holding proximity constant, whereas the contribution of proximity itself would require varying the interpersonal distance; the present study addresses the former. By applying pose estimation to naturalistic video data, Chambers et al. (2019) showed that pairs of pedestrians tended to synchronize their walking movements. Nessler and Gilliland (2010) reported that gait synchronization may occur both intentionally and unintentionally. In their study, forced (intentional) synchronization referred to synchronization produced when a walker was explicitly instructed to match their steps to those of the partner, whereas unforced (unintentional) synchronization arose spontaneously during side-by-side walking without such instruction. They found that, relative to unforced synchronization, forced synchronization led to shorter step length and increased walking speed. The development of gait assistance systems has further leveraged this phenomenon. The WALK-MATE system reproduces interpersonal gait synchronization by generating rhythmic auditory cues through a coupled-oscillator (mutual entrainment) model whose timing continuously and bidirectionally adapts to the user’s gait, rather than imposing a fixed external rhythm; in this way, the system and the user mutually synchronize, as two people do during side-by-side walking. This approach has demonstrated gait stability improvements in patients with PD and hemiplegia and has exhibited a carry-over effect after the intervention (Miyake, 2009; Uchitomi et al., 2013; Uchitomi et al., 2016). This phenomenon also links interpersonal gait synchronization to rhythmic auditory cueing, an established gait therapy in which patients with PD entrain their cadence to an external rhythm such as a metronome (Ghai et al., 2018). Whereas metronome cueing provides a fixed, unidirectional external rhythm, interpersonal gait synchronization involves bidirectional, mutual entrainment between two walkers; how these two forms of rhythmic guidance relate remains largely unexamined.

Despite this evidence, fundamental questions regarding the direct effects of interpersonal gait synchronization on gait asymmetry remain unresolved. Nessler et al. (2015) reported that side-by-side treadmill walking reduced gait asymmetry induced by unilateral ankle loading; nevertheless, their experimental design did not sufficiently distinguish the effects of gait synchronization *per se* from those attributable to side-by-side walking proximity. Furthermore, treadmill walking differs kinematically and kinetically from overground walking (Riley et al., 2007; Alton et al., 1998; Schmitt et al., 2021), limiting the ecological validity of treadmill-based findings in real-world rehabilitation contexts. To date, no study has experimentally isolated the effects of interpersonal gait synchronization on gait asymmetry during overground walking under a controlled asynchronization condition.

To address these gaps, the present study aimed to investigate the effects of interpersonal gait synchronization on gait asymmetry during overground side-by-side walking. Specifically, we examined: (i) whether interpersonal gait synchronization reduces gait asymmetry in individuals with a simulated gait disorder during overground side-by-side walking, and (ii) whether any such reduction persists after synchronization ceases, indicating a short-term carry-over effect. In the present design, the partner adapted to the pseudo-patient rather than the reverse: the pseudo-patient walked naturally and was given no instruction to match steps, whereas the partner was instructed either to walk in synchrony with the pseudo-patient’s gait or to refrain from synchronizing. This asymmetric arrangement reflects the naturalistic rehabilitation setting, in which a therapist walks alongside and adapts to the patient rather than requiring the patient to match an imposed rhythm; from the perspective of the measured participant, synchronization therefore arose without explicit instruction. We did not treat either mode of synchronization as inherently superior to the other. Establishing whether interpersonal gait synchronization, rather than proximity alone, reduces gait asymmetry would help identify the active component of therapist-accompanied walking and inform the design of synchronization-based assistive systems.

## 2 Materials and methods

### 2.1 Study design

This study employed a within-participant design in which gait asymmetry was experimentally induced in one member of each participant pair by applying unilateral ankle loading, a validated method for simulating asymmetric gait disorders in healthy individuals (Nessler et al., 2015; Kwek and Williams, 2021; Skinner and Barrack, 1990). Each pair subsequently performed overground walking side-by-side under two conditions: a synchronization condition and a control asynchronization condition. The asynchronization condition was specifically included to distinguish the effects of gait synchronization from those of side-by-side walking proximity alone. Interpersonal synchronization was verified using the Gait Synchronization Index (GSI) (Zivotofsky et al., 2012), and gait asymmetry was quantified using a validated logarithmic asymmetry index (Plotnik et al., 2007).

### 2.2 Participants

**Figure 1:**
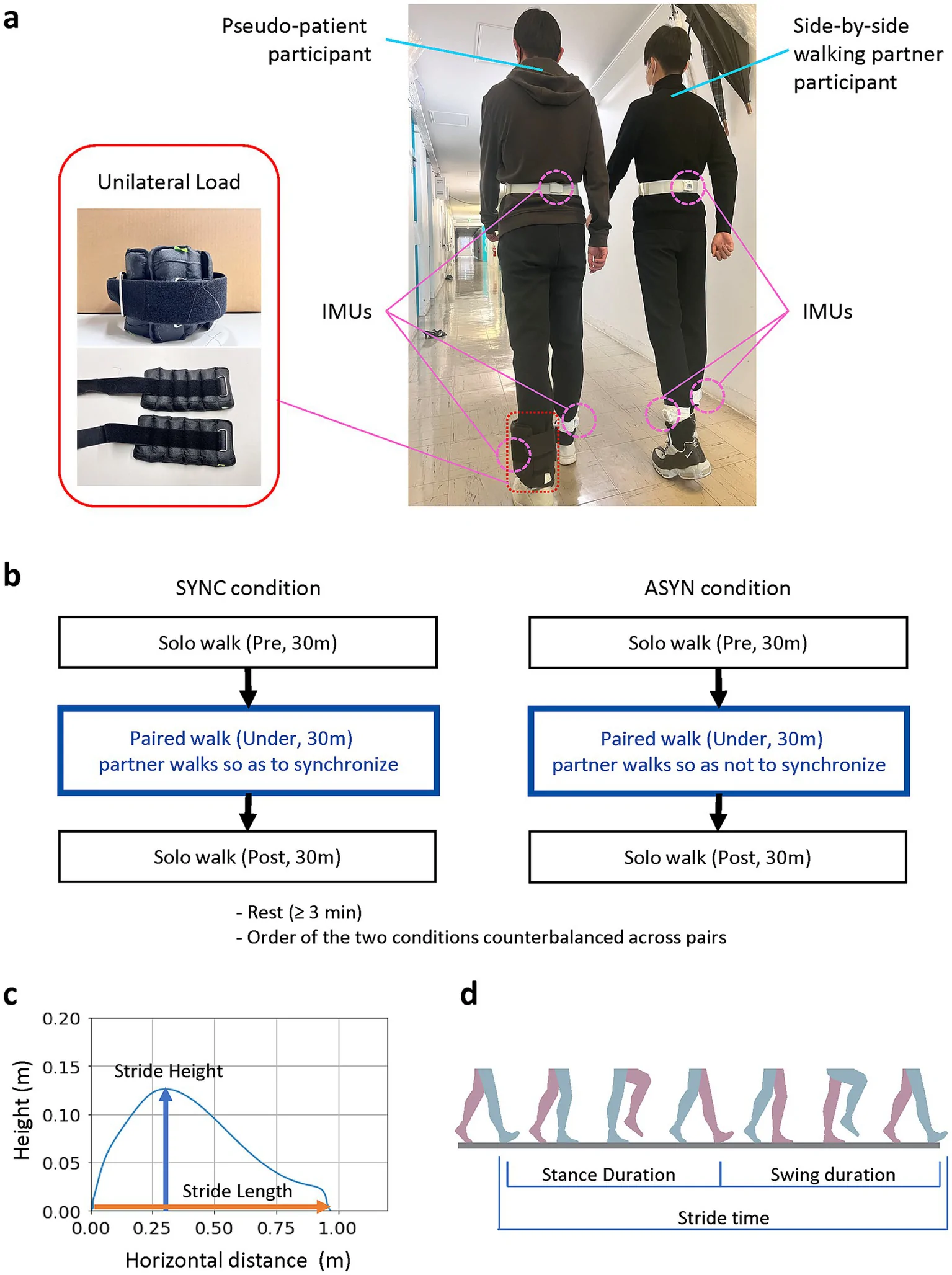
Experimental design and protocol. **(a)** Participant roles and measurement setup: One member of each pair (labeled pseudo-patient) wore a unilateral ankle load, whereas the other member acted as the walking partner; leader lines indicate the correspondence between each label and the participant. Circles indicate the placement of the inertial measurement unit sensors (one on the trunk and one on each ankle), and the inset shows the sandbag used as the unilateral ankle load. **(b)** Walking task structure: Each condition consisted of three sequential 30-m trials [solo pre-trial (Pre), paired walking trial (Under), and solo post-trial (Post)]. The pseudo-patient walked naturally in all trials; under the synchronization condition, the partner walked to synchronize with the pseudo-patient, whereas under the asynchronization condition, the partner walked so as not to synchronize. Each pair completed both conditions, separated by a rest period of at least 3 min, with the order of conditions counterbalanced across pairs. **(c)** Gait parameters illustrated on a side-view foot trajectory: Stride height (maximum foot clearance) and stride length. **(d)** Gait cycle parameters: Stride time, swing duration, and stance duration.

This study included ten pairs (five male–male pairs, three female–female pairs, and two mixed-sex pairs) of healthy adults [12 men and 8 women; mean age: 24.25 ± 1.86 years (range: 21–27 years)]. In each pair, one participant wore the unilateral load and was designated as the pseudo-patient (i.e., a healthy participant with experimentally induced gait asymmetry used to model a gait disorder), whereas the other participant acted as the walking partner (Figure 1a). Pairs were matched such that the difference in lower-limb length between partners did not exceed 10 cm. All participants were healthy individuals with no history of gait disorders.

This study was conducted in accordance with the principles embodied in the Declaration of Helsinki and its amendments and was approved by the Ethics Committee of the Institute of Science Tokyo (approval number: 2024084). Prior to the experiment, written informed consent was obtained from all participants after they received a verbal explanation of the procedure.

### 2.3 Tasks and conditions

Each experimental session involved walking tasks separated by rest periods. A rest period of at least 3 min was provided between conditions and was extended at the participant’s request. Participants were instructed not to talk during the walking trials. Each participant walked at a self-selected pace, and the self-selected paces of the two members of each pair were similar. Each walking task comprised three sequential walking trials: a solo pre-trial (Pre), a paired walking trial (Under), and a solo post-trial (Post) (Figure 1b). In the solo trials, each pseudo-patient continuously walked at a self-selected pace along a straight 30-m corridor, providing baseline (Pre) and post-synchronization (Post) measurements for the short-term carry-over effect analysis. In the paired trial, both participants walked side by side along the same 30-m corridor.

Two paired walking conditions were implemented. In both paired conditions, the pseudo-patient was instructed to walk naturally and was given no instruction regarding synchronization. Under the synchronization condition, the walking partner was instructed to walk in synchrony with the pseudo-patient’s gait. Under the asynchronization condition, the partner was instructed not to synchronize with the pseudo-patient, for example, by varying their step length. No practice trials, metronome, or other external pacing was provided. Each participant pair completed both conditions, with the order being counterbalanced across pairs.

### 2.4 Apparatus

For gait asymmetry induction, a sandbag equivalent to 5% of the pseudo-patient’s body weight was attached to either the left or right ankle. The side of the unilateral ankle load (right or left) was counterbalanced across participants. Before data collection, a 5-min adaptation period was provided to the participants to allow them to habituate to the load, ensuring both participant safety and data stability. Throughout the trial, the sandbag was secured at ankle level, and the participants were instructed to notify the experimenter if the sandbag shifted or detached.

Gait data were recorded using the WALK-MATE Gait Checker system (WALK-MATE LAB Co., Ltd.). This system employs three compact inertial measurement unit sensors (one placed on the trunk and one on each ankle; Figure 1a) and enables continuous stride-by-stride estimation of spatiotemporal gait trajectories during overground walking (Hori et al., 2020; Mao et al., 2021; Uchitomi et al., 2022).

### 2.5 Gait asymmetry index

Five spatiotemporal gait parameters were extracted for analysis: stride height (maximum foot clearance), stride length, stride time, swing duration, and stance duration (Figures 1c,d). For each parameter, gait asymmetry was calculated using the logarithmic asymmetry index proposed by Plotnik et al. (2007), as defined in Equation (1):


$$\mathrm{GA}=100\times |\ln(\bar{x_{l}}/\bar{x_{r}})|$$ (1)

where GA denotes the gait asymmetry index for a given gait parameter, and x_l and x_r represent the mean values of the left- and right-limb parameters, respectively. This index was originally introduced for swing time; in the present study its application was extended to each of the five spatiotemporal parameters, and it was computed separately for every parameter. A gait asymmetry value of 0 indicated perfect bilateral symmetry, whereas larger values denoted greater asymmetry. A previous study reported a swing time asymmetry of approximately 7 after unilateral ankle loading in healthy individuals (Nessler et al., 2015). For each walking trial, the first two and the last two steps were excluded to remove the gait-initiation and gait-termination phases, and all remaining steps, corresponding to the steady-state walking period, were included in the analysis.

### 2.6 Gait synchronization index

Because the degree of synchronization achieved might vary across pairs and trials, the degree of interpersonal gait synchronization was quantified objectively using the Gait Synchronization Index (GSI), which applies to gait the Shannon entropy-based synchronization index proposed by Tass et al. (1998) and was introduced for side-by-side walking by Zivotofsky et al. (2012, 2018). The GSI ranges from 0 (a uniform relative-phase distribution, indicating no synchronization) to 1 (a constant relative phase, indicating perfect synchronization). The onset of each gait cycle was identified from the output of the WALK-MATE Gait Checker system. A trial was considered “synchronized” if GSI > 0.20; otherwise, it was deemed to be “asynchronized” (Tass et al., 1998). The GSI was defined as shown in Equation (2):


$$\mathrm{GSI}=1-\frac{\sum_{\mathrm{RP}}P_{x_{\mathrm{RP}}}\ln\frac{1}{P_{x_{\mathrm{RP}}}}}{\ln M}$$ (2)

where (Pikovsky et al., 2001); RP denotes the relative phase (in degrees) between the two walkers, t0 is the time at which the gait cycle of one participant began, t2 is the time at which the same participant’s gait cycle ended (i.e., the onset of that participant’s next gait cycle), so that t2 − t0 is the cycle duration, and t1 is the time of the corresponding gait-cycle onset of the other participant occurring within the interval [t0, t2]. The relative phases obtained across all *N* gait cycles of a trial were binned into *M* equal-width intervals spanning 0–360°, and PxRP was estimated as the relative frequency nx/N, where nx is the number of cycles in bin x. The number of bins was determined as *M* = exp (0.626 + 0.4 ln(*N* − 1)) (Otnes and Enochson, 1972).

### 2.7 Statistical analysis

Prior to inferential testing, the normality of gait asymmetry distribution under each condition was assessed using the Shapiro–Wilk test. To ensure consistency, nonparametric tests were used because non-normality was detected in two out of six conditions for stride height (Pre under the synchronization condition: *W* = 0.803, *p* = 0.016; Post under the synchronization condition: *W* = 0.752, *p* = 0.004). Overall differences across time points (Pre, Under, and Post) for each condition were examined using Friedman’s test. *Post-hoc* pairwise comparisons were conducted using the Wilcoxon signed-rank test with Bonferroni correction across nine comparisons per parameter. For each of the five gait parameters, these nine comparisons comprised three within-condition comparisons under the synchronization condition (Pre vs. Under, Pre vs. Post, and Under vs. Post), the corresponding three comparisons under the asynchronization condition, and three between-condition comparisons at each time point (Pre, Under, and Post). The significance level was set at *α* = 0.05, and the Bonferroni-corrected threshold was therefore *α* = 0.05/9 = 0.0056; uncorrected *p* values are reported throughout and were compared against this corrected threshold. Effect sizes were calculated as *r* = |*Z*|/sqrt(*N*), where *Z* is the standardized test statistic of the Wilcoxon signed-rank test and *N* is the number of pairs (*N* = 10); values of 0.10, 0.30, and 0.50 were interpreted as small, medium, and large effects, respectively (Cohen, 1988). The effect size obtained for stride height (*r* = 0.979) therefore exceeds the threshold for a large effect by a considerable margin and is accordingly described as very large. In addition, the homogeneity of variance of gait asymmetry between conditions was examined using Levene’s test based on medians, and the stride-to-stride variability of stride length was quantified as the coefficient of variation (CV) and compared between conditions using the Wilcoxon signed-rank test. These two analyses were exploratory and were not included in the Bonferroni-corrected family of nine comparisons. All statistical analyses were performed using R version 4.5.3 (R Foundation for Statistical Computing, Vienna, Austria).

## 3 Results

### 3.1 Gait synchronization verification

**Figure 2:**
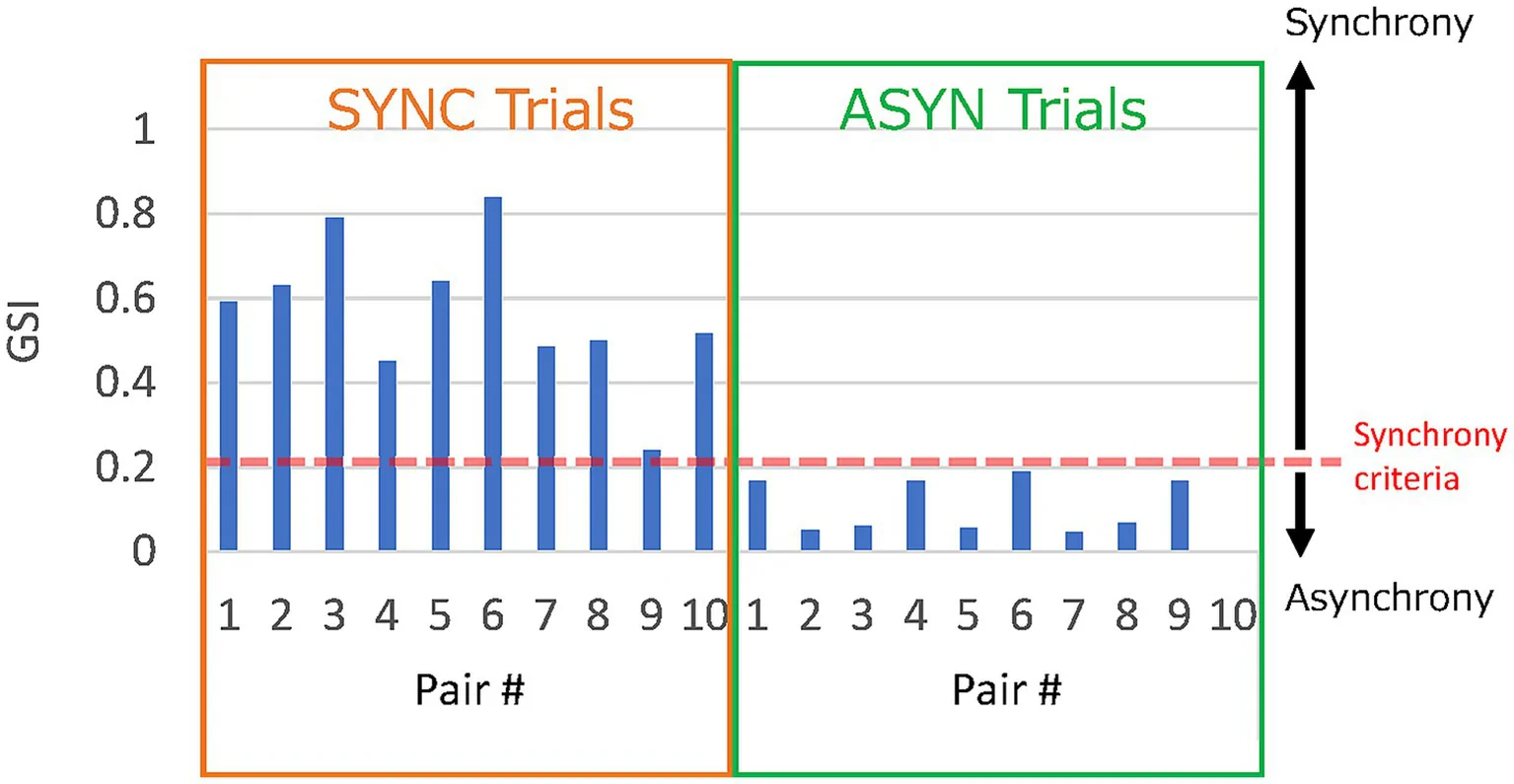
Gait Synchronization Index (GSI) results for all ten participant pairs. Left panel (orange border): Under the synchronization condition, all GSI values exceeded the synchrony criterion of 0.20 (red dashed line), confirming successful synchronization. Right panel (green border): Under the asynchronization condition, all GSI values fell below 0.20, confirming asynchronized walking. The synchrony criterion of GSI > 0.20 was adopted from Tass et al. (1998) and Zivotofsky et al. (2012). The double-headed arrow on the right denotes the direction of increasing synchrony (upward) and asynchrony (downward).

The GSI values of all experimental pairs under both conditions are presented in Table 1 and Figure 2. All GSI values under the synchronization condition exceeded the synchronization threshold of 0.20 (range: 0.241–0.838), indicating the achievement of synchronized walking in all pairs. Conversely, all GSI values under the asynchronization condition fell below 0.20 (range: 0.000–0.189), confirming that asynchronized walking was maintained throughout. These results validated the experimental manipulation and verified that the two conditions were effectively differentiated.

**Table 1:** Gait Synchronization Index (GSI) values for all 10 participant pairs under the synchronization and asynchronization conditions.

Pair #	Synchronization condition	Asynchronization condition
1	0.592	0.167
2	0.631	0.052
3	0.789	0.060
4	0.450	0.167
5	0.641	0.057
6	0.838	0.189
7	0.484	0.046
8	0.499	0.068
9	0.241	0.166
10	0.516	0.000

All synchronization condition values exceed the synchrony threshold of 0.20; all asynchronization condition values fall below it.

### 3.2 Gait asymmetry: stride height

**Figure 3:**
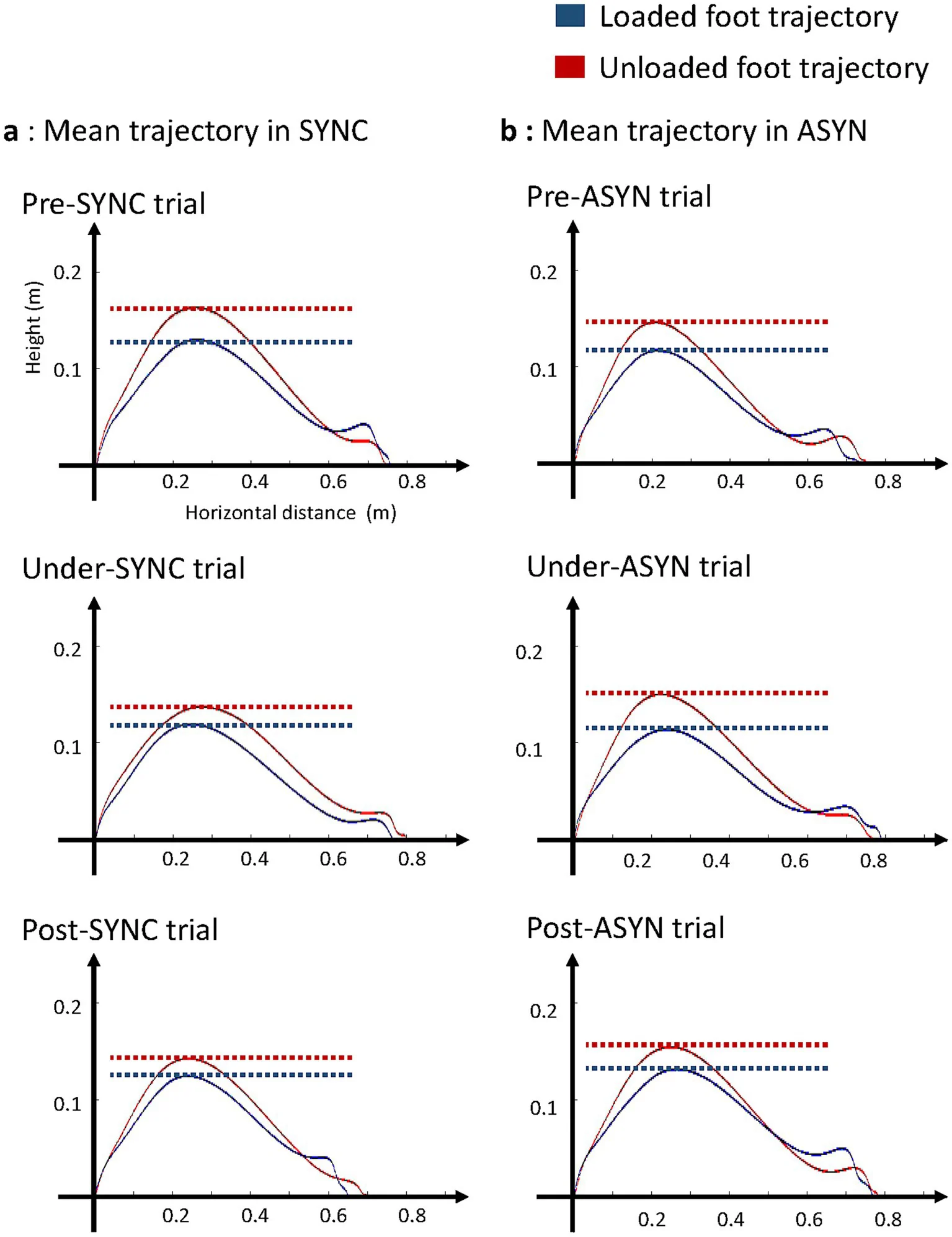
Representative mean foot trajectories of one pseudo-patient. **(a)** Mean foot trajectories under the synchronization condition and **(b)** under the asynchronization condition, each shown for the Pre-, Under-, and Post-trials. Each trace is the mean trajectory across the analyzed strides of that trial for the loaded foot (blue) and the unloaded foot (red). Dotted horizontal lines indicate the mean stride height of each foot.

Representative foot trajectory data from one pseudo-patient are presented in Figure 3. Under the synchronization condition (Figure 3a), the difference in mean stride height between the loaded and unloaded feet was clearly visible in the Pre trial, became diminished in the Under trial, and remained reduced in the Post trial. Under the asynchronization condition (Figure 3b), the difference between the loaded and unloaded feet was not diminished in the Under trial and remained at a level comparable to that of the Pre trial in the Post trial.

**Figure 4:**
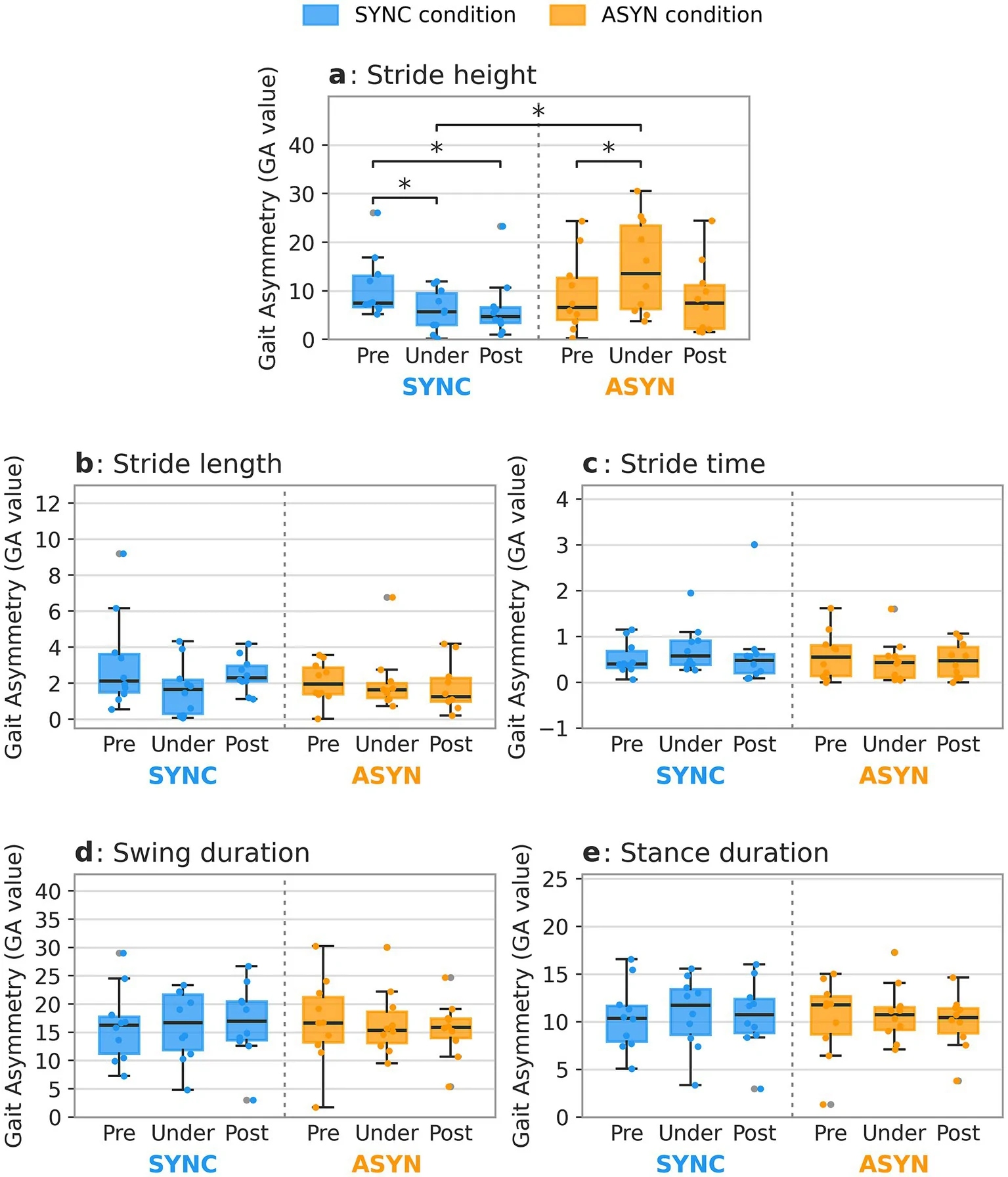
Gait asymmetry results across five spatiotemporal gait parameters under synchronization (blue) and asynchronization (orange) conditions. Each panel depicts gait asymmetry values at Pre (baseline), Under (during paired walking), and Post (after paired walking) time points. Boxes represent the median and interquartile range, whereas whiskers denote 1.5 times the interquartile range; individual data points are overlaid. For clarity, brackets are shown only for the *post-hoc* Wilcoxon signed-rank comparisons that reached significance at the Bonferroni-corrected threshold (*uncorrected *p* < 0.0056); Non-significant comparisons are not annotated and are reported in full in Table 2. **(a)** Stride height: Gait asymmetry significantly decreased from Pre to Under and from Pre to Post in the synchronization condition, and a significant difference between the synchronization and asynchronization conditions was observed at the Under time point. **(b)** Stride length: No significant difference was observed under either condition. **(c)** Stride time: No significant difference was observed under either condition. **(d)** Swing duration: No significant difference was observed under either condition. **(e)** Stance duration: No significant difference was observed under either condition.

Statistical results for all five gait parameters are presented in Figure 4 and Table 2. Descriptive statistics (median [interquartile range] and mean (standard deviation)) for all five parameters under both conditions and at all three time points are provided in Supplementary Table S1. Friedman’s test indicated a significant overall effect of time point on stride height under the synchronization condition (*χ*^2^ = 15.200, df = 2, *p* < 0.001) and a significant effect under the asynchronization condition (*χ*^2^ = 9.600, df = 2, *p* = 0.008). *Post-hoc* Wilcoxon signed-rank test with Bonferroni correction (corrected alpha = 0.0056) revealed that stride height was significantly reduced from Pre to Under in the synchronization condition (*W* = 0, *p* = 0.002, *r* = 0.979), indicating an immediate effect of synchronization. This reduction was maintained from Pre to Post in the synchronization condition (*W* = 0, *p* = 0.002, *r* = 0.979), implying a short-term carry-over effect. Furthermore, in the Under trial, stride height asymmetry was significantly lower under the synchronization condition than under the asynchronization condition (*W* = 0, *p* = 0.002, *r* = 0.979), confirming the specificity of the synchronization effect. In addition to the test statistics, the magnitude of these differences was estimated using Hodges–Lehmann median differences with distribution-free 95% confidence intervals: 3.85 (95% CI 2.25–9.72) for Pre versus Under and 3.60 (95% CI 2.60–6.66) for Pre versus Post under the synchronization condition, and 9.22 (95% CI 4.18–13.95) for the between-condition comparison in the Under trial. Under the asynchronization condition, stride height asymmetry increased significantly from Pre to Under (*W* = 0, *p* = 0.002, *r* = 0.979), with all ten pairs showing an increase, and returned to a level comparable to baseline in the Post trial (*p* = 0.432); the corresponding medians [interquartile ranges] were 6.63 [4.03–12.65] at Pre, 13.58 [6.27–23.47] at Under, and 7.49 [2.23–11.19] at Post. No other pairwise comparisons reached significance after Bonferroni correction. Notably, stride height asymmetry did not significantly change from Pre to Post under the asynchronization condition.

**Table 2:** Results of Friedman’s test and *post-hoc* Wilcoxon signed-rank test with Bonferroni correction for gait asymmetry across five parameters.

Analysis	Comparison	SH	SL	ST	SwD	StD
Friedman	Synchronization condition (Pre/Under/Post)	*χ*^2^ = 15.200, *p* < 0.001^***^	*χ*^2^ = 0.800, *p* = 0.670 n.s.	*χ*^2^ = 4.200, *p* = 0.122 n.s.	*χ*^2^ = 0.600, *p* = 0.741 n.s.	*χ*^2^ = 0.800, *p* = 0.670 n.s.
	Asynchronization condition (Pre/Under/Post)	*χ*^2^ = 9.600, *p* = 0.008^**^	*χ*^2^ = 1.400, *p* = 0.497 n.s.	*χ*^2^ = 0.800, *p* = 0.670 n.s.	*χ*^2^ = 0.200, *p* = 0.905 n.s.	*χ*^2^ = 0.600, *p* = 0.741 n.s.
Under the synchronization condition	Pre vs. Under	*W* = 0, *p* = 0.002^*^, *r* = 0.979	*W* = 14, *p* = 0.193 n.s., *r* = 0.411	*W* = 16, *p* = 0.275 n.s., *r* = 0.345	*W* = 26, *p* = 0.922 n.s., *r* = 0.031	*W* = 19, *p* = 0.432 n.s., *r* = 0.249
	Pre vs. Post	*W* = 0, *p* = 0.002^*^, *r* = 0.979	*W* = 23, *p* = 0.695 n.s., *r* = 0.124	*W* = 23, *p* = 0.695 n.s., *r* = 0.124	*W* = 22, *p* = 0.625 n.s., *r* = 0.155	*W* = 22, *p* = 0.625 n.s., *r* = 0.155
	Under vs. Post	*W* = 27, *p* = 1.000 n.s., *r* = 0.000	*W* = 12, *p* = 0.131 n.s., *r* = 0.478	*W* = 14, *p* = 0.193 n.s., *r* = 0.411	*W* = 21, *p* = 0.557 n.s., *r* = 0.186	*W* = 20, *p* = 0.492 n.s., *r* = 0.217
Under the asynchronization condition	Pre vs. Under	*W* = 0, *p* = 0.002^*^, *r* = 0.979	*W* = 21, *p* = 0.557 n.s., *r* = 0.186	*W* = 19, *p* = 0.432 n.s., *r* = 0.249	*W* = 25, *p* = 0.846 n.s., *r* = 0.062	*W* = 20, *p* = 0.492 n.s., *r* = 0.217
	Pre vs. Post	*W* = 19, *p* = 0.432 n.s., *r* = 0.249	*W* = 24, *p* = 0.770 n.s., *r* = 0.093	*W* = 17, *p* = 0.322 n.s., *r* = 0.313	*W* = 22, *p* = 0.625 n.s., *r* = 0.155	*W* = 25, *p* = 0.846 n.s., *r* = 0.062
	Under vs. Post	*W* = 5, *p* = 0.020 n.s., *r* = 0.738	*W* = 23, *p* = 0.695 n.s., *r* = 0.124	*W* = 27, *p* = 1.000 n.s., *r* = 0.000	*W* = 27, *p* = 1.000 n.s., *r* = 0.000	*W* = 20, *p* = 0.492 n.s., *r* = 0.217
Between Conditions	Pre: synchronization vs. asynchronization conditions	*W* = 18, *p* = 0.375 n.s., *r* = 0.281	*W* = 19, *p* = 0.432 n.s., *r* = 0.249	*W* = 27, *p* = 1.000 n.s., *r* = 0.000	*W* = 23, *p* = 0.695 n.s., *r* = 0.124	*W* = 27, *p* = 1.000 n.s., *r* = 0.000
	Under: synchronization vs. asynchronization conditions	*W* = 0, *p* = 0.002^*^, *r* = 0.979	*W* = 17, *p* = 0.322 n.s., *r* = 0.313	*W* = 15, *p* = 0.232 n.s., *r* = 0.378	*W* = 19, *p* = 0.432 n.s., *r* = 0.249	*W* = 27, *p* = 1.000 n.s., *r* = 0.000
	Post: synchronization vs. asynchronization conditions	*W* = 20, *p* = 0.492 n.s., *r* = 0.217	*W* = 9, *p* = 0.064 n.s., *r* = 0.585	*W* = 22, *p* = 0.625 n.s., *r* = 0.155	*W* = 21, *p* = 0.557 n.s., *r* = 0.186	*W* = 22, *p* = 0.625 n.s., *r* = 0.155

Uncorrected *p* values are reported throughout. Effect size *r* = |*Z*|/sqrt(*N*). SH, stride height; SL, stride length; ST, stride time; SwD, swing duration; StD, stance duration. Friedman’s test was evaluated at alpha = 0.05 (^***^p < 0.001; ^**^p < 0.01). For the *post-hoc* Wilcoxon signed-rank tests, a Bonferroni correction was applied across the nine comparisons per parameter, giving a corrected significance threshold of alpha = 0.05/9 = 0.0056; ^*^denotes comparisons significant at this threshold. n.s., not significant.

### 3.3 Other gait parameters

Friedman’s test indicated no significant overall effect on the remaining four parameters (namely, stride length, stride time, swing duration, and stance duration) under either the synchronization or asynchronization condition (all *p* > 0.12; Table 2). Accordingly, no *post-hoc* comparisons were conducted for these parameters.

## 4 Discussion

### 4.1 Principal findings

The principal findings were threefold. First, stride height asymmetry was substantially reduced during synchronized walking relative to baseline. Second, this reduction persisted after synchronization had ceased, indicating a short-term carry-over effect. Third, stride height asymmetry during the Under trial was lower under the synchronization than the asynchronization condition. No significant effects were observed on the asymmetry of stride length, stride time, swing duration, or stance duration under either condition.

### 4.2 Comparison with previous studies and novelty

The present findings extend the results reported by Nessler et al. (2015), who reported that side-by-side treadmill walking reduced the gait asymmetry induced by unilateral ankle loading. However, Nessler et al. did not isolate the role of synchronization *per se* from the general effect of walking near another person. The present study addresses this limitation directly by comparing the synchronization and asynchronization conditions while holding all other variables constant. The difference in gait asymmetry between the synchronization and asynchronization conditions during the Under trial therefore indicates that gait synchronization, rather than side-by-side walking proximity alone, is the active component underlying the reduction in gait asymmetry.

The present findings also extend previous treadmill-based results to an overground walking context, which more closely reflects real-world rehabilitation and daily ambulation (Riley et al., 2007; Alton et al., 1998; Schmitt et al., 2021). The carry-over effect observed during the Post trial, in which stride height asymmetry remained significantly low compared with baseline even after the partner had departed, has not been previously reported for interpersonal gait synchronization. This finding aligns with the carry-over effect documented for WALK-MATE-based interventions (Uchitomi et al., 2016), suggesting that brief interpersonal synchronization may produce short-term retention of the reduced asymmetry. Because the Post trial was conducted immediately after the paired walking trial, the present design can address only a short-term carry-over effect, and the persistence of this effect beyond the immediate post-trial period remains to be established. A possible mechanism underlying this short-term retention, namely short-term motor learning or sensorimotor adaptation induced by synchronized walking, is considered in Section 4.4.

### 4.3 Specificity of the synchronization effect

A notable finding observed under the asynchronization condition was the marked increase in stride height asymmetry from Pre to Under. The variability of stride height asymmetry during the Under trial was significantly greater under the asynchronization than the synchronization condition (Levene’s test based on medians, *W* = 10.50, *p* = 0.005; SD 4.27 vs. 9.75), although the increase in variability from Pre to Under within the asynchronization condition was not itself significant (*p* = 0.281). In addition, the stride-to-stride variability of stride length in the partner was significantly higher under the asynchronization than the synchronization condition (Wilcoxon signed-rank test, *W* = 1, *p* = 0.004, *r* = 0.913; median CV 0.056 vs. 0.085), whereas the corresponding variability of the pseudo-patient did not differ significantly between conditions (*W* = 15, *p* = 0.232). Because proximity was held constant across the two conditions, the greater variability of stride height asymmetry under the asynchronization condition indicates that walking beside a partner influenced the pseudo-patient’s gait, and that the direction of this influence depended on the gait characteristics of the partner, consistent with bidirectional interpersonal gait coupling. The increased stride-to-stride variability observed in the partner under the asynchronization condition is consistent with the instruction given in that condition. What is specific to synchronization is therefore the reduction of gait asymmetry, rather than the presence of an effect per se. The contrast between the decrease in gait asymmetry under the synchronization condition (Pre to Under: approximately −45%) and the increase in gait asymmetry under the asynchronization condition (Pre to Under: approximately +60%) underscores the bidirectional influence of interpersonal gait coupling on individual gait performance. These percentages refer to changes in mean asymmetry; the corresponding per-pair relative changes, which corroborate the reduction under the synchronization condition, are reported in Supplementary Table S2.

### 4.4 Mechanistic considerations

The observed improvements in stride height asymmetry may be explained by the mutual entrainment of gait rhythm through audiovisual exchange of footstep and body movement information between partners (Muto and Miyake, 2011). Mutual gait synchronization dynamically stabilizes walking movement (Miyake, 2009). As indicated by the present findings, such stabilization may extend to bilateral gait asymmetry reduction. In the present context, the unilateral ankle load primarily constrained foot lift-off on the loaded side, producing the observed stride height asymmetry. During synchronized walking, the rhythmic coupling between partners might have facilitated compensatory adjustments in the pseudo-patient’s gait, enabling partial recovery of foot clearance on the loaded side.

The short-term carry-over effect observed during the Post trial may reflect short-term motor learning or sensorimotor adaptation induced by synchronized walking experience. Rhythmic synchronization-based movement has been proposed to engage motor learning processes through auditory–motor coupling, reinforcing internal timing mechanisms and promoting gait pattern stabilization (Miyake, 2009; Uchitomi et al., 2013). Indeed, in patients with neurological gait disorders, rhythm-based interventions have been associated with gait improvements that persist beyond the training period (Krakauer et al., 2019). Although the present participants were healthy adults with simulated asymmetry, brief exposure to interpersonal gait synchronization may engage similar motor learning processes, which could contribute to the observed short-term carry-over effect. However, the neural substrates of this short-term carry-over effect remain to be elucidated in future studies employing neuroimaging or neurophysiological measures.

### 4.5 Clinical implications

The present findings have potential implications for gait rehabilitation. Brief synchronization during overground walking may reduce gait asymmetry and produce a short-term carry-over effect, suggesting that therapist–patient side-by-side walking, a common clinical practice, may derive its effectiveness at least in part from interpersonal gait synchronization. These results also provide empirical support for the design of gait assistance systems that leverage interpersonal gait synchronization, such as the WALK-MATE series (Miyake, 2009; Uchitomi et al., 2013; Uchitomi et al., 2016) and avatar-based gait guidance systems (Miller et al., 2022). Although the present study included healthy individuals with simulated gait asymmetry, the principles demonstrated herein may be applicable to the rehabilitation of patients with neurological gait disorders, including post-stroke patients (Patterson et al., 2008) or those with PD. It should be noted, however, that the mechanism of asymmetry differs between the present model and clinical populations, and that the applicability of these principles is likely to depend on the residual adaptive capacity of each patient (see Section 4.6).

### 4.6 Limitations

This study has some limitations. First, the sample size of ten pairs may limit the statistical power and generalizability of the findings. We did not compute *post-hoc* power, because power calculated from the observed effect is a deterministic function of the *p* value and provides no additional information (Hoenig and Heisey, 2001). Instead, a sensitivity analysis indicated that, with ten pairs and *α* = 0.05, the present design had approximately 80% power to detect a large within-pair effect (d_z ≈ 1.0); the observed effects for stride height were of this magnitude or larger (d_z = 0.97 for Pre vs. Under and d_z = 1.58 for Pre vs. Post, with all ten pairs changing in the same direction). Effect size point estimates obtained from small samples should nevertheless be interpreted with caution, and replication in larger samples is required.

Second, the participants were healthy young adults with artificially induced gait asymmetry rather than clinical patients. While unilateral ankle loading is a validated model for simulating gait asymmetry (Nessler et al., 2015; Kwek and Williams, 2021), the findings may not fully generalize to patients with neurological gait disorders, who exhibit more complex and variable asymmetry profiles (Patterson et al., 2008). Third, the mechanism by which the unilateral ankle load induces asymmetry differs from that of clinical gait disorders, in which asymmetry arises from spasticity, muscle weakness, or impaired rhythm generation rather than from a purely mechanical constraint; the applicability of the present findings to patients therefore depends on the extent of their residual adaptive capacity. Fourth, limb dominance was not recorded in this study. Because the load was applied unilaterally, limb dominance is a potential confounding factor; the loading side was counterbalanced across participants, but future studies should record limb dominance and examine its interaction with the loading side and with whether the loaded limb is closer to or farther from the partner. For the same reason, a sub-analysis of these factors could not be performed here.

Fifth, proximity itself was not manipulated. Although proximity was held constant across the two conditions, evaluating the role of proximity would require varying the interpersonal distance, and the present design therefore cannot exclude a general effect of proximity on gait. Sixth, although the order of the two conditions was counterbalanced across pairs, a 5-min adaptation period preceded the walking tasks, and each condition had its own Pre baseline; thus, learning or familiarization effects cannot be entirely excluded. Future studies should incorporate a washout period between conditions.

Seventh, gait asymmetry was quantified using stride-based parameters rather than step-based parameters, because these were the parameters provided by the measurement system used in this study; step-based parameters, which may offer complementary and more limb-specific information, were therefore not examined and should be analyzed in future work. In addition, the first and last two steps of each trial were excluded to preserve enough steady-state strides for analysis. However, gait-initiation and gait-termination phases may extend beyond two steps; future replication studies should therefore consider excluding a larger number of steps (for example, the first and last five steps) to more conservatively remove these transient phases. Eighth, we did not include a third condition in which the pseudo-patient was instructed to synchronize with the partner. Only the partner was instructed to synchronize, because the aim of this study was to examine synchronization that arises without explicit instruction to the measured participant, mirroring the naturalistic rehabilitation setting in which a therapist adapts to the patient. A condition in which the patient is instructed to match an imposed rhythm is conceptually closer to cueing-based therapies and was therefore beyond the present scope, although it warrants direct comparison in future work. Furthermore, trajectory-based measures such as cross-correlation of foot trajectories were not analyzed; both represent avenues for future work. Ninth, the swing duration asymmetry observed here at baseline (median 16.2 under the synchronization condition) was higher than the value of approximately 7 reported under comparable unilateral ankle loading on a treadmill (Nessler et al., 2015). This difference may be related to differences in the walking conditions, namely overground walking at a self-selected pace in the present study versus treadmill walking at an imposed, pair-averaged speed in the previous study. The factors underlying this difference remain to be clarified in future studies. Finally, the gait characteristics of the partner may also be relevant. We therefore examined the gait asymmetry of the partner during the paired trials and found no significant difference in stride height asymmetry between the pseudo-patient and the partner (Supplementary Table S3). The asymmetry of the partner during solo walking was not measured in the present study, and this aspect warrants further investigation in future studies. Future studies should address these limitations by recruiting larger and clinically diverse samples and by examining the effects of synchronization duration on gait asymmetry outcomes.

## 5 Conclusion

This study provides experimental evidence that interpersonal gait synchronization reduces stride height asymmetry during overground side-by-side walking and produces a short-term carry-over effect that persists immediately after synchronization ceases; the associated effect size was very large. By explicitly comparing a synchronization condition and a control asynchronization condition, this study revealed that the reduction in gait asymmetry was specific to interpersonal gait synchronization and was not produced by side-by-side walking proximity alone. These findings provide empirical support for the development of synchronization-based gait assistance systems and suggest that interpersonal gait synchronization may serve as a basis for effective gait rehabilitation interventions. Future studies should examine these effects in clinical populations with neurological gait disorders and investigate the neural mechanisms underlying the carry-over effect.

## Data Availability

The raw data supporting the conclusions of this article will be made available by the authors, without undue reservation.
