# Supplementary material for: Interpersonal gait synchronization reduces gait asymmetry and induces a carry-over effect during overground side-by-side walking
Source: Front Psychol. 2026 Sep 9;17:1892679. doi: 10.3389/fpsyg.2026.1892679 (PMC13597440; doi:10.3389/fpsyg.2026.1892679)
Supplement: Supplementary file 1 [file Table_1.DOCX]

**Supplementary Material**

**1 Supplementary Data**

**1.1 Descriptive statistics for all gait asymmetry parameters**

Supplementary Table S1 reports the descriptive statistics for the gait asymmetry (GA) index for all five spatiotemporal gait parameters under the synchronization and asynchronization conditions at the Pre, Under, and Post time points (n = 10 pairs). GA was computed using the logarithmic asymmetry index defined in Equation 1 of the main text and is dimensionless. The corresponding inferential results are reported in Table 2 of the main text.

**1.2 Relative change in stride height asymmetry**

Section 4.3 of the main text describes the change in stride height asymmetry as a decrease of approximately 45% under the synchronization condition and an increase of approximately 60% under the asynchronization condition. These figures refer to the change in the *mean* GA between time points. To confirm that this effect does not depend on the choice of summary statistic, Supplementary Table S2 also reports the median of the per-pair relative change, computed by first calculating the relative change for each pair and then taking the median across pairs.

**1.3 Gait asymmetry of the partner during the paired trials**

Supplementary Table S3 reports the gait asymmetry of stride height for the pseudo-patient and the walking partner during the paired (Under) trials under both conditions (n = 10 pairs). The values for the pseudo-patient correspond to the Under rows for stride height in Supplementary Table S1. The two members of each pair were compared using the Wilcoxon signed-rank test. The gait asymmetry of the partner during the solo trials was not measured, because only the pseudo-patient was recorded in those trials.

**2 Supplementary Tables**

**Table S1.** Descriptive statistics of gait asymmetry for all five spatiotemporal gait parameters (n = 10 pairs).

| **Parameter** | **Condition** | **Time point** | **Median [IQR]** | **Mean (SD)** |
| --- | --- | --- | --- | --- |
| Stride height | Synchronization | Pre | 7.49 [6.69-13.10] | 10.87 (6.52) |
|  |  | Under | 5.72 [2.99-9.52] | 5.99 (4.27) |
|  |  | Post | 4.75 [3.48-6.63] | 6.62 (6.49) |
|  | Asynchronization | Pre | 6.63 [4.03-12.65] | 9.35 (7.92) |
|  |  | Under | 13.58 [6.27-23.47] | 15.00 (9.75) |
|  |  | Post | 7.49 [2.23-11.19] | 8.51 (7.48) |
| Stride length | Synchronization | Pre | 2.12 [1.49-3.63] | 3.15 (2.67) |
|  |  | Under | 1.66 [0.32-2.18] | 1.68 (1.51) |
|  |  | Post | 2.31 [2.11-2.98] | 2.49 (0.98) |
|  | Asynchronization | Pre | 1.96 [1.40-2.88] | 2.07 (1.12) |
|  |  | Under | 1.64 [1.19-2.01] | 2.10 (1.74) |
|  |  | Post | 1.26 [1.00-2.29] | 1.80 (1.37) |
| Stride time | Synchronization | Pre | 0.41 [0.32-0.68] | 0.53 (0.35) |
|  |  | Under | 0.58 [0.39-0.91] | 0.73 (0.51) |
|  |  | Post | 0.48 [0.20-0.62] | 0.65 (0.86) |
|  | Asynchronization | Pre | 0.56 [0.15-0.81] | 0.59 (0.53) |
|  |  | Under | 0.43 [0.10-0.58] | 0.48 (0.47) |
|  |  | Post | 0.48 [0.13-0.77] | 0.49 (0.39) |
| Swing duration | Synchronization | Pre | 16.24 [11.26-17.76] | 16.23 (6.63) |
|  |  | Under | 16.74 [11.92-21.65] | 16.18 (6.18) |
|  |  | Post | 16.97 [13.61-20.45] | 16.85 (6.76) |
|  | Asynchronization | Pre | 16.67 [13.23-21.24] | 16.92 (7.77) |
|  |  | Under | 15.39 [13.11-18.63] | 16.71 (5.95) |
|  |  | Post | 15.88 [13.99-17.44] | 15.52 (5.10) |
| Stance duration | Synchronization | Pre | 10.39 [7.92-11.68] | 10.47 (3.57) |
|  |  | Under | 11.76 [8.64-13.43] | 10.93 (3.80) |
|  |  | Post | 10.78 [8.84-12.39] | 10.66 (3.73) |
|  | Asynchronization | Pre | 11.78 [8.70-12.65] | 10.39 (4.13) |
|  |  | Under | 10.74 [9.16-11.51] | 10.89 (3.03) |
|  |  | Post | 10.46 [8.80-11.41] | 9.98 (2.90) |

IQR, interquartile range; SD, standard deviation. Gait asymmetry is expressed using the units of the logarithmic asymmetry index defined in Equation 1 of the main text. Pre, baseline solo walking; Under, paired walking; Post, solo walking immediately after paired walking.

**Table S2.** Relative change in stride height asymmetry, expressed as the change in group mean and as the median of the per-pair relative change (n = 10 pairs).

| **Comparison** | **Change in mean GA** | **Median of per-pair relative change [95% CI]** |
| --- | --- | --- |
| Synchronization, Pre vs. Under | −44.9% | −42.1% [−82.9%, −17.9%] |
| Synchronization, Pre vs. Post | −39.1% | −44.8% [−69.5%, −20.8%] |
| Asynchronization, Pre vs. Under | +60.5% | +52.4% [+19.8%, +492.7%] |

Relative change was computed as (GA*after* − GA*before*) / GA*before* × 100; negative values indicate a reduction in asymmetry. "Change in mean GA" is the change in the group mean and corresponds to the figures quoted in Section 4.3 of the main text. "Median of per-pair relative change" was obtained by computing the relative change separately for each pair and then taking the median across pairs, with a distribution-free 95% confidence interval based on order statistics. For the asynchronization condition, this per-pair median is strongly influenced by pairs with near-zero baseline asymmetry, and the wide confidence interval should be interpreted with caution.

**Table S3.** Gait asymmetry of stride height for the pseudo-patient and the walking partner during the paired (Under) trials (n = 10 pairs).

| **Condition** | **Participant** | **Median [IQR]** | **Mean (SD)** | **Wilcoxon signed-rank test** |
| --- | --- | --- | --- | --- |
| Synchronization | Pseudo-patient | 5.72 [2.99-9.52] | 5.99 (4.27) | W = 9, p = 0.064, r = 0.585 |
|  | Partner | 15.44 [5.40-22.06] | 15.28 (10.48) |  |
| Asynchronization | Pseudo-patient | 13.58 [6.27-23.47] | 15.00 (9.75) | W = 23, p = 0.695, r = 0.124 |
|  | Partner | 17.44 [9.91-20.05] | 16.85 (9.55) |  |

IQR, interquartile range; SD, standard deviation. Gait asymmetry is expressed using the units of the logarithmic asymmetry index defined in Equation 1 of the main text. Under, paired walking. The pseudo-patient wore the unilateral ankle load, whereas the partner did not. W, Wilcoxon signed-rank test statistic; r, effect size calculated as |Z|/sqrt(N), where N is the number of pairs (N = 10); uncorrected p values are reported. These comparisons were exploratory and were not included in the Bonferroni-corrected family of nine comparisons reported in the main text.
